# Acceptability of Prenatal Screening Tests Among Expectant Mothers in India: Insights and Implications for Public Health

**DOI:** 10.7759/cureus.61246

**Published:** 2024-05-28

**Authors:** Sangeetha Arumugam, Sri Sowmya Kalluri, Vijayan Sharmila, Nandha Kumar Subbiah, Akarsh Mocherla, Jyoti Kulkarni, Joy A Ghoshal

**Affiliations:** 1 Anatomy, All India Institute of Medical Sciences, Mangalagiri, Mangalagiri, IND; 2 Obstetrics and Gynecology, Siddhartha Medical College, Vijayawada, IND; 3 Obstetrics and Gynecology, All India Institute of Medical Sciences, Mangalagiri, Mangalagiri, IND

**Keywords:** public health policy, maternal screening, prenatal genetic testing, non-invasive prenatal test, nipt, prenatal ultrasound, nuchal translucency, prenatal care awareness, down's syndrome, chromosomal aneuploidies

## Abstract

Introduction: Prenatal screening tests are essential for preventing common genetic disorders, yet their acceptability among pregnant women in India remains unexplored. This study aims to investigate the acceptability of prenatal screening tests and their correlation with demographic characteristics among pregnant women in India.

Methods: A cross-sectional study was conducted at a tertiary care, public hospital, involving 200 pregnant women. Data were collected through a self-administered questionnaire assessing demographic information and the acceptability of prenatal screening tests. Statistical analysis included chi-square tests and logistic regression.

Results: Most participants demonstrated adequate acceptability toward prenatal screening tests, with 73% scoring above the threshold. Factors associated with higher acceptability included younger maternal age, second-trimester gestational age, higher education, salaried employment, and urban residence. However, factors such as parity, consanguinity, mode of conception, and family history of genetic disease showed no significant associations.

Conclusion: The study highlights positive attitudes toward prenatal screening tests among pregnant women in India, particularly among younger, more educated, and urban populations. These findings emphasize the need for targeted interventions to enhance awareness and accessibility of prenatal screening, ultimately contributing to the reduction of the genetic disorder burden in India.

## Introduction

Prenatal screening tests are offered to all pregnant women for the prevention of common genetic disorders. Screening tests include ultrasonography, maternal serum assays, and most recently, maternal plasma fetal cell-free fetal DNA, which can detect aneuploidy, microdeletion, and copy number variants [1]. Prenatal diagnosis is indicated for women who are positive at the screening stage or have other risk factors, such as advanced maternal age, family history of a genetic disorder, or abnormal ultrasound findings [2]. The insights gained from prenatal testing have not only been utilized to reduce the incidence of genetic diseases through the termination of affected pregnancies but also to prevent adverse outcomes for both the fetus and the mother [3,4].

Several countries have adopted national policies for prenatal testing programs [5]. In India, a population-based government scheme for prenatal screening is not available [6]. Approximately 21,400 children with Down syndrome, 9000 with beta-thalassemia, and 5200 with sickle cell disease are born in India every year [7,8]. Hence, experts have recommended a nationwide, government-funded program aimed at preventing genetic disorders by including prenatal testing as a part of routine antenatal care [6,9].

Prevention of genetic diseases through prenatal testing depends primarily on the awareness and acceptability of the available tests by the expectant mothers. Analyzing their mindset would provide a reference point for the introduction of prenatal genetic testing programs [10]. Very little is known about women’s acceptability toward prenatal genetic screening. While numerous studies have explored attitudes, awareness, and acceptance of prenatal testing in various populations and regions globally, there is a scarcity of literature addressing the acceptability of tests among pregnant women in India [11-14]. Therefore, this study aims to provide insights into the acceptability of prenatal screening tests for genetic disorders among pregnant women in India and to examine how these factors correlate with demographic characteristics.

## Materials and methods

Study design

This cross-sectional study was conducted at a tertiary care, public hospital over a six-month period in 2023 by the Genetic Counseling Unit, Department of Anatomy, and Department of Obstetrics and Gynecology at the All India Institute of Medical Sciences, Mangalagiri, Mangalagiri, India. Ethical clearance was obtained from the Institutional Ethical Committee of All India Institute of Medical Sciences, Mangalagiri (AIIMS/MG/IEC/2022-23/240) prior to the study. Two hundred pregnant women visiting the Department of Obstetrics and Gynecology for antenatal care were included in the study. Due to the absence of similar studies in India, an assumption was made that acceptability for prenatal screening tests among pregnant women was at 50%. With a study power of 80%, a confidence level of 95%, and a precision of 7%, the sample size was calculated as 196, rounded up to 200. Pregnant women of all age groups willing to participate were included, while those unwilling or who had already responded to the questions were excluded.

Data collection instrument

Data collection utilized a self-administered questionnaire available in both English and the local language, Telugu. Translation into the local language was performed by a native language expert. The questionnaire consisted of two parts. Part 1 gathered demographic information such as maternal age, gestational age, parity, consanguinity, family history of genetic disease, educational status, and place of residence. Part 2 was an acceptability assessment form comprising seven questions designed to assess acceptability regarding prenatal screening tests among pregnant women. All the questions were designed based on the literature search and were validated by subject experts [11-14]. The questionnaire underwent pilot testing among 20 pregnant women not included in the study, and minor changes were made to improve the clarity of the questions.

Statistical analysis

Acceptability scores were derived from five questions, with the correct answers (yes) scored as 1, and incorrect (no) or unanswered (don't know) questions receiving a score of 0. A score of 3 or above indicated adequate acceptability, while a score of 2 or below indicated inadequate acceptability. Data were tabulated and coded using Microsoft Excel (Microsoft Corporation, Redmond, United States), and statistical analysis was conducted using GraphPad Prism software (GraphPad Software, San Diego, United States). Categorical variables were summarized as frequencies and proportions. The chi-square test assessed associations between demographic variables and acceptability levels, while logistic regression analysis identified factors associated with acceptability. A p-value of ≤0.05 was considered statistically significant.

## Results

A total of 200 pregnant women, attending the hospital for antenatal care, participated in the study, yielding a response rate of 100%. Table 1 presents the demographic characteristics of the 200 women who participated in the study.

**Table 1:** Socio-demographic characteristics of participants

	Socio-demographic characteristics of the participants		n	%
1	Age of the pregnant women	<21 yrs.	40	20
		22-25 yrs.	72	36
		25-29 yrs.	69	34.5
		>30 yrs.	19	9.5
2.	Gestational age of fetus	First trimester	53	26.5
		Second trimester	61	30.5
		Third trimester	86	43
3	Parity	Primigravida	88	44
		Multigravida	112	56
4	Consanguinity	Consanguineous	26	13
		Non-consanguineous	174	87
5	Mode of conception	Natural conception	189	94.5
		Assisted conception	11	5.5
6	Family history of genetic disease	Yes	21	10.5
		No	179	89.5
7	Highest education	Degree	107	53.5
		High school	51	25.5
		Primary school	42	21
8	Employment	Salaried	26	13
		Self-employed	14	7
		Unemployed	160	80
9	Socioeconomic status	Upper class	03	1.5
		Middle class	169	84.5
		Below poverty line	28	14
10	Residence	Urban	71	35.5
		Semi-urban	63	31.5
		Rural	66	33

**Figure 1:**
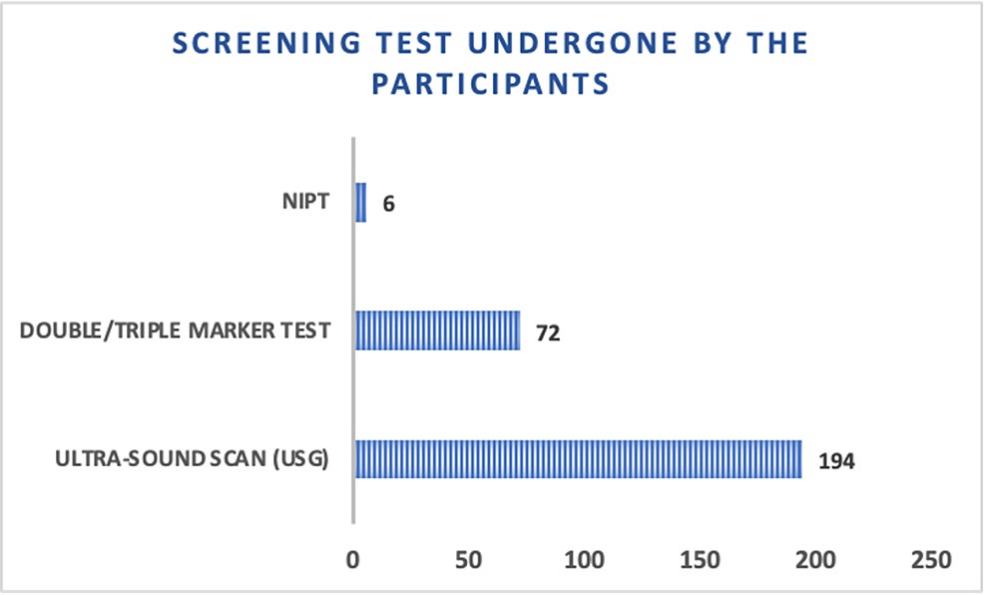
Bar diagram showing the distribution of prenatal screening tests that the participants have undergone (both in the current pregnancy or earlier pregnancies) NIPT: noninvasive prenatal testing test

**Figure 2:**
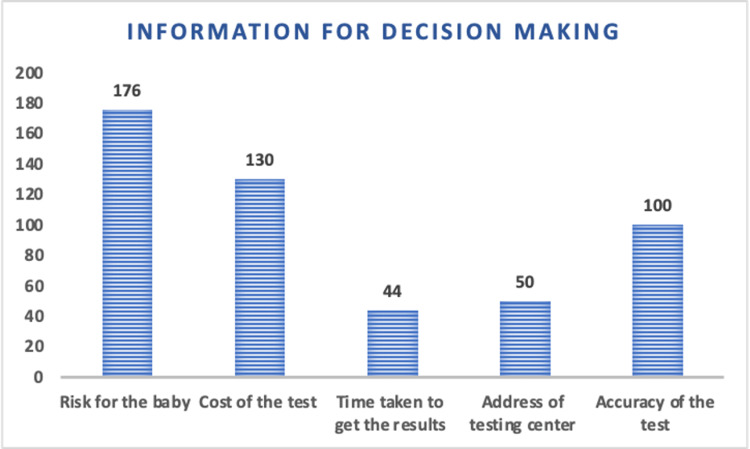
Bar diagram showing the distribution of the information that the participants would like to know before deciding to undergo a confirmatory test (following a positive prenatal screening result)

Both in the current pregnancy or earlier pregnancies, most participants had undergone ultrasound scans (194), while some had undergone the double/triple test (72). Very few (6) had undergone noninvasive prenatal testing test (NIPT) (Figure 1). Prior to deciding to undergo confirmatory testing, the majority expressed a desire to know about the risk for the baby (176). Additionally, a significant proportion of participants wanted information on the cost of the test (130), and the accuracy of the test (100), followed by inquiries about the address of the testing centers (50), and the turnaround time (44) (Figure 2).

The acceptability levels of prenatal screening tests among pregnant women were assessed through a questionnaire comprising five questions. The majority of participants expressed willingness to undergo prenatal screening tests if advised by a doctor, with 83% responding affirmatively, while only 8% answered negatively, and 9% were unsure. Additionally, a significant proportion (76%) indicated readiness to visit specialized centers for confirmatory tests in case of abnormal ultrasound scans and blood test results. Furthermore, 69% of participants expressed interest in discussing prenatal screening and confirmatory tests with genetic counselors or healthcare workers. A noteworthy finding was that 84% of respondents believed that awareness about these tests should be made compulsory for all women in early pregnancy. Moreover, a considerable percentage (85%) showed support for a country-wide prenatal screening program to detect genetic disorders in fetuses, with only 4% opposing the idea (Table 2). The acceptability score, derived from the women's responses to the questionnaire, revealed adequate acceptability among 146 (73%) participants and inadequate acceptability among 54 (27%).

**Table 2:** Acceptability levels of prenatal screening tests among pregnant women

	Acceptability questionnaire	Yes n (%)	No n (%)	Don’t know n (%)
1	If advised by the doctor, are you willing to undergo prenatal screening tests during the present or next pregnancy?	166 (83)	16 (8)	18 (9)
2.	If Ultra-Sound (USG) scan and blood test results are abnormal, are you willing to visit specialized centers to undergo confirmatory tests?	152 (76)	26 (13)	22 (11)
3	Would you like to discuss with genetic counselor or other healthcare workers to know more about prenatal screening and confirmatory tests used to detect genetic disorders?	138 (69)	42 (21)	20 (10)
4	Do you think that awareness on prenatal screening and confirmatory test should be made compulsory for all women in early pregnancy?	168 (84)	10 (5)	22 (11)
5	Do you support for a country-wide prenatal screening program for pregnant mothers to detect genetic disorders in the fetus?	170 (85)	8 (4)	22 (11)

The association between demographic factors and the acceptability of prenatal screening tests among pregnant women was examined using chi-square analysis. Among the demographic variables assessed, age showed a statistically significant association with acceptability levels (χ^2^ = 11.578, p = 0.008). Pregnant women aged 22-25 years demonstrated the highest percentage of adequate acceptability (37.6%), while those above 30 years showed the lowest (12.4%). Additionally, the gestational age of the fetus exhibited a significant association with acceptability (χ^2^ = 6.616, p = 0.037), with higher acceptability observed in the third trimester (44.5%). Education level also displayed a significant association (χ^2^ = 10.692, p = 0.004), with a higher proportion of women with a degree demonstrating adequate acceptability (60.2%). Employment status (χ^2^ = 11.100, p = 0.003) and residence (χ^2^ = 20.104, p = 0.00004) also showed statistically significant associations with acceptability levels. These findings suggest that demographic factors play a role in influencing the acceptability of prenatal screening tests among pregnant women (Table 3).

**Table 3:** Association between demographic factors and acceptability of prenatal screening tests among pregnant women

	Socio-demographic factor	Acceptability of prenatal screening test				Chi-sq	p-value
		Adequate		Inadequate			
		n	%	n	%		
1	Age of the pregnant women					11.578	0.008
	<21 yrs.	22	15.0	18	33.3		
	22-25 yrs.	55	37.6	17	31.6		
	25-29 yrs.	51	35.0	18	33.3		
	>30 yrs.	18	12.4	01	01.8		
2	Gestational age of fetus					6.616	0.037
	First trimester	33	22.6	20	37.0		
	Second trimester	51	35.0	10	18.5		
	Third trimester	62	42.4	24	44.5		
3	Parity					1.455	0.228
	Primigravida	68	46.5	20	37.0		
	Multigravida	78	53.4	34	63.0		
4	Consanguinity					2.045	0.153
	Consanguineous	22	15.0	04	07.4		
	Non-consanguineous	124	85.0	50	92.6		
5	Mode of conception					0.459	0.497
	Natural conception	137	94.0	52	96.3		
	Assisted conception	09	06.0	02	03.7		
6	Family history of genetic disease					0.121	0.727
	Yes	16	10.9	05	09.2		
	No	130	89.1	49	90.8		
7	Highest education					10.692	0.004
	Degree	88	60.2	19	35.2		
	High school	30	20.5	21	38.8		
	Primary school	28	19.3	14	26.0		
8	Employment					11.100	0.003
	Salaried	26	17.8	00	00.0		
	Self-employed	10	09.0	04	07.4		
	Unemployed	110	75.2	50	92.6		
9	Socioeconomic status					1.497	0.473
	Upper class	03	02.0	00	00.0		
	Middle class	124	85.0	45	83.3		
	Below poverty line	19	13.0	09	16.7		
10	Residence					20.104	0.00004
	Urban	41	28.1	30	55.5		
	Semi-urban	58	39.7	05	09.2		
	Rural	47	32.2	19	35.3		
	Total	146	73.0	54	27.0		

The logistic regression analysis identified several factors associated with adequate acceptability of prenatal screening tests among pregnant women. Younger maternal age was positively associated, with women aged 22-25 years (odds ratio (OR) = 2.85, 95% CI: 1.33-6.08, p = 0.007) and 25-29 years (OR = 2.82, 95% CI: 1.30-6.11, p = 0.009) having higher odds of adequate acceptability compared to younger (<21 years) women. Second-trimester gestational age was also a positive correlate, with these women having 2.38 times greater odds (95% CI: 1.01-5.60, p = 0.047) of adequate acceptability versus the first trimester. Higher educational attainment was important, with women holding a university degree having 3.60 times greater odds (95% CI: 1.64-7.91, p = 0.001) of adequate acceptability compared to those with a primary school education. Salaried employment was also a significant predictor, with salaried women having over six times higher odds (OR = 6.32, 95% CI: 1.95-20.53, p = 0.002) of adequate acceptability versus unemployed women. Finally, urban residence emerged as a strong positive correlate, with urban women having 4.46 times greater odds (95% CI: 2.13-9.35, p < 0.001) of adequate acceptability compared to rural residents. Other factors like parity, consanguinity, mode of conception, and family history were not significantly associated with acceptability.

## Discussion

Prenatal screening tests play a crucial role in identifying common genetic disorders and facilitating appropriate interventions to prevent adverse outcomes for both the fetus and the mother. As a result, the decision-making process is often intricate and emotionally challenging. Our study investigated the acceptability of prenatal screening tests among pregnant women in India and examined the correlation between acceptability and demographic characteristics.

The findings from various studies conducted in different regions provide valuable insights into the acceptability of prenatal screening tests among pregnant women. In Lagos, a significant majority (81.3%) expressed a belief in the necessity of offering these tests to all expectant mothers, with a substantial proportion (75.1%) indicating a willingness to pursue further diagnostic testing if initial screening results raised concerns [15]. Similarly, studies in Greece, Ibadan, and Sokoto, and reported high levels of acceptability, with percentages ranging from 68% to 95.2% [13,16,17]. In the research conducted in Saudi Arabia, participants expressed their endorsement of government-operated facilities aimed at providing genetic testing or screening services to the country's citizens [18]. Our study, conducted among 200 participants, revealed that 73% demonstrated adequate acceptability scores, aligning with the positive attitudes observed in previous research. Moreover, 76% of respondents in our study expressed readiness to visit specialized centers to undergo confirmatory tests if screening test results are suggestive, 84% advocated for mandatory awareness initiatives, and 85% supported the implementation of a nationwide screening program in India. Our result is consistent with previous studies that have reported similar findings highlighting an optimistic outlook toward prenatal testing among pregnant women.

The effectiveness of these disease prevention strategies depends not just on the knowledge and perspectives of professionals but also on the awareness and willingness of the recipients. Studies have reported significant associations between awareness levels and factors such as age, trimester of pregnancy, and education level [19,20]. The association between demographic factors and the acceptability of prenatal screening tests in the present series indicate that younger maternal age had higher odds of adequate acceptability toward prenatal screening tests. This suggests that younger women may be more receptive to preventive measures and proactive healthcare practices during pregnancy compared to older women. Similarly, women in the second trimester of pregnancy exhibited higher acceptability levels, indicating that as the pregnancy progresses, women may become more open to undergoing prenatal screening tests, possibly due to increased awareness and engagement with healthcare providers.

Acceptability scores were positively correlated with the educational status of the participants [15,16,21]. In our study, educational attainment emerged as a significant predictor of acceptability, with women holding a university degree demonstrating greater acceptance of prenatal screening tests. This underscores the role of education in enhancing health literacy and promoting informed decision-making regarding prenatal care. Positive attitudes toward genetic testing were correlated with demographic factors such as higher levels of income [22,23]. In the current series, employment status and urban residence were associated with higher odds of adequate acceptability. Salaried women and those residing in urban areas exhibited a greater willingness to undergo prenatal screening tests, suggesting that access to resources and healthcare infrastructure may influence acceptability levels. However, several demographic factors such as parity, consanguinity, mode of conception, and family history did not show significant associations with acceptability. This highlights the need for targeted interventions to promote awareness and enhance acceptability among diverse demographic groups.

Our study has several implications for public health policy and practice. First, the findings underscore the importance of incorporating prenatal screening tests into routine antenatal care, particularly among younger women, those in the second trimester of pregnancy, and those with higher educational attainment. Second, efforts to increase awareness and accessibility to prenatal screening tests, particularly in rural areas and among marginalized populations, are essential to ensure equitable access to preventive healthcare services.

Limitations of the study

One limitation of our study is the potential for selection bias, as the participants were recruited from a single tertiary care public hospital, which may not fully represent the broader population of pregnant women in India. Additionally, the study relied on self-reported data, which introduces the possibility of response bias. Moreover, the study did not evaluate the acceptability of prenatal screening tests among expectant fathers, which could provide valuable insights into family decision-making processes regarding prenatal care. Furthermore, the cross-sectional design of the study limits the ability to establish causal relationships between demographic factors and acceptability levels. Finally, the study did not explore cultural or religious factors that may influence attitudes toward prenatal testing, which could be addressed in future research to provide a more comprehensive understanding of acceptability among diverse populations.

## Conclusions

Our study highlights the positive attitude of pregnant women in India toward prenatal screening tests, with significant support for government-led initiatives. Factors such as younger age, higher education, salaried employment, and urban residence were associated with greater acceptability. These findings underscore the importance of targeted interventions to increase awareness and accessibility to prenatal screening, particularly among marginalized populations. Further research is needed to explore cultural and religious influences on attitudes toward prenatal testing and assess the impact of interventions on screening uptake and health outcomes. Overall, our study contributes valuable insights to inform public health policies aimed at reducing the burden of genetic disorders in India.
